# Synergistic Corrosion Inhibition of Mild Steel in Acidic Media by a Benzimidazole–Thiophene Ligand and Its Metal Complexes: A Multi-Technique Electrochemical Approach

**DOI:** 10.3390/ma18194545

**Published:** 2025-09-30

**Authors:** Mariya Kadiri, Majid Driouch, Ibissam Elaaraj, Ayoub Tanji, Afafe Elabbadi, Mohammed Fahim, Mouhcine Sfaira, Hendra Hermawan

**Affiliations:** 1 Laboratory of Engineering, Modelling and Systems Analysis (LIMAS), Faculty of Sciences, University Sidi Mohamed Ben Abdellah (USMBA), P.O. Box 1796, Fez-Atlas 30000, Morocco; mariya.kadiri.1@ulaval.ca (M.K.); majid.driouch@usmba.ac.ma (M.D.); afafe.elabbadi@usmba.ac.ma (A.E.); mouhcine.sfaira@usmba.ac.ma (M.S.); 2 Department of Mining, Metallurgical and Materials Engineering, Laval University, Quebec City, QC G1V 0A6, Canada; ayoub.tanji@cegeptr.qc.ca; 3 Laboratory of Innovative Materials and Biotechnologies of Natural Resources, Faculty of Sciences, Moulay Ismail University, P.O. Box 11201, Meknes 50000, Morocco; 4 Quebec Metallurgy Center, Trois-Rivières, QC G9A 5E1, Canada

**Keywords:** corrosion, organometallic inhibitor, electrochemical approach

## Abstract

This study investigates the corrosion inhibition efficiency of [2-(thiophen-2-yl)-1-(thiophen-2-ylmethyl)-1H-benzo[d]imidazole] and its Zn and Cu complexes for mild steel in 1.0 M HCl. The ligand was selected for its non-toxic profile and high electron density, favoring strong adsorption onto the metal surface. Electrochemical methods, including EIS, PDP, LPR, and CASP, were employed to evaluate the inhibitors’ performance. The results showed a significant decrease in corrosion current density and increased polarization resistance, with the Zn complex achieving the highest inhibition efficiency (93.8%). EIS fitting confirmed the formation of a protective film with high charge transfer and film resistance. Surface analyses by SEM and EDS revealed smoother steel morphology and inhibitor adsorption. XPS confirmed the presence of Fe^3+^, Zn^2+^and Cu^2+^ oxides, as well as all active inhibitor elements on the surface, supporting a mixed inhibition mechanism. The enhanced performance of the metal complexes is attributed to synergistic effects between the metal ions and the heterocyclic ligand, offering a promising strategy for the design of effective and environmentally friendly corrosion inhibitors.

## 1. Introduction

Corrosion remains a critical environmental and industrial challenge, posing serious risks to infrastructure durability and economic stability worldwide. Its by-products, such as rust, not only degrade structural materials but also contribute to aquatic and soil contamination, thereby impacting both engineered and natural ecosystems [1]. Globally, the financial burden of corrosion is staggering: the total cost is estimated at approximately USD 2.5 trillion annually, accounting for nearly 3.4% of the global GDP. According to data from the Financial Times and NACE International, corrosion-related damage to steel structures, industrial facilities, and public infrastructure costs the global economy over USD 3.0 billion each year [2,3]. Mild steel (MS), a material widely used in industrial applications due to its mechanical properties and cost-effectiveness, is particularly susceptible to corrosion in aggressive environments, especially those containing chloride or sulfate ions. Thus, the development of efficient and sustainable corrosion mitigation strategies is of paramount importance from both economic and safety standpoints [4].

Among the various corrosion prevention techniques, the use of corrosion inhibitors has garnered significant attention due to their high efficiency and low dosage requirements [5,6]. These inhibitors, when introduced in small concentrations into a corrosive medium, can substantially reduce the rate of metal degradation by forming protective layers at the metal-electrolyte interface.

Organic inhibitors dominate current research and industrial applications, primarily due to their structural versatility and ability to adsorb onto metal surfaces through electron-rich functional groups. Compounds containing heteroatoms such as nitrogen, sulfur, and oxygen exhibit strong adsorption via lone pair electrons and π–electron systems, thereby impeding anodic and/or cathodic corrosion reactions. Numerous studies have demonstrated that the incorporation of additional heterocycles or heteroatoms in the inhibitor structure enhances inhibition efficiency. Representative classes include thiols [7], amines [8], Schiff bases [9,10], ionic liquids [11,12], surfactants [13], and pharmaceutical molecules [14], all of which have shown promising performance in acidic media.

Recent trends in inhibitor research have shifted toward hybrid systems combining organic and inorganic functionalities [15,16]. Organic inhibitors generally function through surface adsorption, forming stable, compact films, whereas inorganic inhibitors such as chromates, phosphates, and molybdates typically form insoluble precipitates or passive oxide layers that physically block the access of corrosive agents. The integration of these two mechanisms has led to the development of synergistic formulations with superior barrier properties, long-term stability, and tailored functionality for specific environments. Computational modeling methods, such as density functional theory (DFT) [17,18,19], alongside advanced surface characterization methods like XPS and SEM-EDS, have further enabled the rational design of next-generation inhibitors with improved environmental compatibility and targeted performance. Despite these advances, designing high-efficiency, non-toxic [20,21], and environmentally friendly corrosion inhibitors, especially those based on transition metals like copper [15,22], zinc [23] remains a vital objective in materials protection research.

In the present study, we investigate the corrosion inhibition performances of novel organometallic systems based on the compound [2-(thiophen-2-yl)-1-(thiophen-2-ylmethyl)-1H-benzo[d]imidazole], both in its free ligand form and complexed with Zn^2+^ and Cu^2+^ ions [24]. This ligand was selected due to its non-toxic profile and structurally rich framework, which includes nitrogen and sulfur atoms known for their strong metallic surfaces affinity as confirmed by Elaaraj I. et al. [24]. The fused thiophene-benzimidazole moiety offers extended conjugation and electron-donating ability, favoring adsorption and the formation of a stable inhibitor layer. By incorporating Zn and Cu into the organic framework, the study aims to explore the role of metal–ligand coordination in enhancing the inhibition efficiency, providing deeper insight into how specific metal centers influence adsorption strength, electronic structure, and passivation behavior.

To thoroughly assess the inhibition efficiency and behavior of these compounds, we employed a suite of electrochemical techniques: electrochemical impedance spectroscopy (EIS), potentiodynamic polarization (PDP), linear polarization resistance (LPR), and constant amplitude signal perturbation (CASP). CASP, in particular, offers unique advantages for corrosion analysis, as it captures the dynamic evolution of corrosion processes under controlled current perturbations [25,26,27]. This multi-technique approach enables both a comprehensive and nuanced understanding of the inhibition mechanisms at play. The experimental sequence was carefully designed to accommodate the destructive or non-destructive nature of each technique, ensuring data integrity and allowing cross-validation of results.

This comparative study provides a systematic evaluation of the impact of metal ion incorporation within the organic ligand structure on corrosion inhibition performance. It also highlights the individual and complementary strengths of various electrochemical methods for characterizing inhibitor behavior. The findings are expected to contribute to the development of advanced, environmentally friendly organometallic corrosion inhibitors with broad applicability across industrial sectors.

## 2. Materials and Methods

### 2.1. Materials

**Figure 1:**
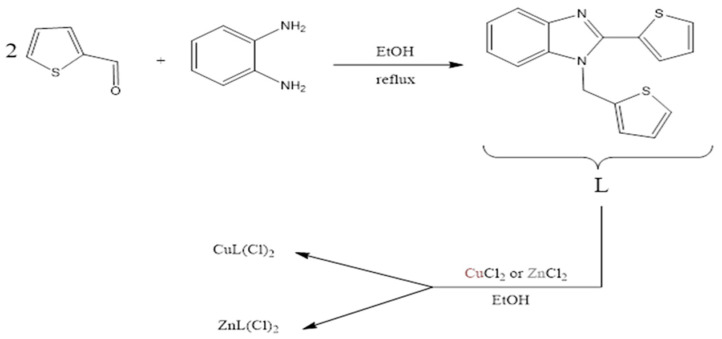
Synthesis of ligand and its complexes Zn(II), Cu(II).

MS specimens used in this study had the following compositions: 2.00 wt.% Mg, 0.50 wt.% Fe, 0.50 wt.% Mn, 0.40 wt.% Si, 0.15 wt.% Cu, 0.10 wt.% Zn, and 0.30 wt.% Cr; they were employed as the working electrodes. The specimens were embedded in epoxy resin with only one surface (1.0 cm^2^) being exposed to the solution in all electrochemical experiments. Before measurements, the surface of MS was polished with 100, 400, 800, 1200, and 2000 emery papers successively then degreased with ethanol, and finally dried by compressed air. The aggressive solution was prepared by diluting hydrochloric acid 37% with deionized water. The inhibition compounds used in this study were the ligand 2-(thiophen-2-yl)-1-(thiophen-2-ylmethyl)-1H-benzo[d]imidazole (L), synthesized by the condensation of thiophene-2-carboxaldehyde with ortho-phenylenediamine, and its Cu(II) and Zn(II) complexes prepared in ethanol as the solvent, as shown in Figure 1. The test solutions were prepared by dissolving the three compounds in 1.0 M HCl to achieve a concentration of 1 × 10^−3^ M.

### 2.2. Electrochemical Measurements

Electrochemical experiments were carried out in the conventional three-electrode cell with a platinum counter electrode (CE) and an Ag/AgCl/3 M KCl coupled to a fine Luggin capillary as the reference electrode. All electrochemical measurements were carried out using potentiostat Biologic SP150 conducted with EC-Lab software version 11.12 (Biologic, Seyssinet-Pariset, France). Before each test, the electrode was immersed in the test solution at the open circuit potential (OCP) for 60 min at 298 K to reach a quasi-stable state of the system. The EIS measurement was recorded in a frequency range from 100 kHz to 100 mHz with amplitude of 10 mV and 10 points per decade. Using the CASP technique requires the choice of a low EIS frequency, so the frequency of 0.1 Hz is low enough to measure polarization resistance and according to the results of EIS, the electrolyte resistance is negligible compared with the polarization resistance. LPR measurement was recorded in scan rate of 1 mV/s from −25 mV to +25 mV around the corrosion potential *E*_corr_. The PDP curves were obtained with a sweep rate of 1 mV/s in the range of potential begin from −250 mV to +250 mV.

### 2.3. Morphology and Surface Analysis

Surface observation was conducted on the specimens using a scanning electron microscope (SEM, Quanta 250 FEI, Hillsboro, OR, USA). Oxide film compositions were characterized, using an X-ray photoelectron spectroscope (XPS, PHI 5600–ci spectrometer, Physical Electronics, Chanhassen, MN, USA) in high resolution and angle resolved modes (AR–XPS) with an analyzed area of 0.5 mm^2^. The spectra were acquired, utilizing a standard aluminum X ray source (Kα = 1486.6 eV) with charge compensation, while high resolution spectra were recorded, using a standard magnesium (Kα = 1253.6 eV).

## 3. Results and Discussion

### 3.1. Electrochemical Evaluation

**Figure 2:**
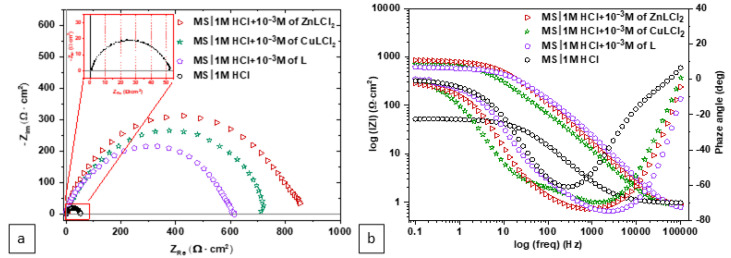
EIS diagrams of MS in 1.0 M HCl with and without inhibitors L, CuLCl_2_ and ZnLCl_2_: (**a**) Nyquist plots; (**b**) Bode plots.

The impedance data are recorded at *E*_corr_ = d*E*_ocp_/dt→0, corresponding to the quasi stationarity of the system, reached after a stabilization of one hour. The Nyquist and/or Bode diagrams, representing EIS data, are given in Figure 2. A detailed inspection of the Nyquist diagrams for the MS/1.0 M HCl system, both in the absence and the presence of the ligand and its complexes, reveals a single, broad, and flattened loop across the entire scanned frequency range, presumably attributed to a single, unequivocal time constant, as evidenced by numerous research studies in the field [28]. The diameter of these loops increases significantly in the presence of the ligand and its complexes when compared to the HCl solution alone, with the largest diameters observed for the complexes, denoted hereafter ZnLCl_2_ and CuLCl_2_, respectively [29]. This indicates the formation of a protective film onto the MS surface, which is further reinforced by the incorporation of metal ions into the ligand’s organic structure. The non-centered nature of the loops suggests non-ideal interfacial behavior, likely caused by complex adsorption/desorption kinetics, surface inhomogeneities, or roughness induced during the corrosion process [30].

The interface being inhomogeneous, it acts like a distribution of time constants, resulting in deviations from the ideal behavior of a capacitor. Typically, these deviations are modeled using constant-phase elements (CPEs) instead of ideal capacitors, to reflect frequency dispersion [31]. The impedance of CPE is:

(1) $$Z_{CPE}=\frac{1}{{Q\times \left(j\omega \right)}^{n}}$$

where Q is the admittance of CPE, ω is the angular frequency, n represents the fitting exponent that ranges from 0 to 1, and j represents the imaginary unit.

In contrast, the analysis of the data in the Bode representation, where the frequency dependences of both the amplitude and the phase angle are more explicit, reveals the presence of two time constants in the case of ZnLCl_2_ and CuLCl_2_. However, this feature becomes concealed in the case of L and the uninhibited solution. Here, between the low frequency (LF) and high frequency (HF) asymptotes, a single transition slope (Δ(*log|Z|*))/(Δ(*log f*)) is clearly observed, accompanied by a single minimum of the phase angle. According to the literature, these results strongly suggest the existence of one constant time [32,33]. However, as indicated in other studies [15], the presence of a single slope (Δ(*log|Z|*))/(Δ(*log f*)) and a single “inverse Gaussian (Φ(Z) vs. log *f*) in the Bode plot does not preclude the possibility of multiple time constants that are poorly distinguished and insufficiently separated in frequency [34].

**Figure 3:**
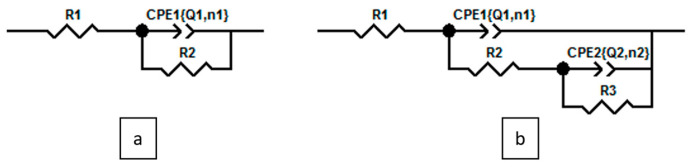
Equivalent electrical circuits models: (**a**) Randles circuit R_1_ + CPE_1_/R_2_; (**b**) Two time constants circuit R_1_ + CPE_1_/(R_2_ + CPE2/R_3_).

Based on these interpretations, and to better evaluate the quality of the experimental data fitting, two equivalent electrical circuit (EEC) models were employed for simulation, as shown in Figure 3.

**Figure 4:**
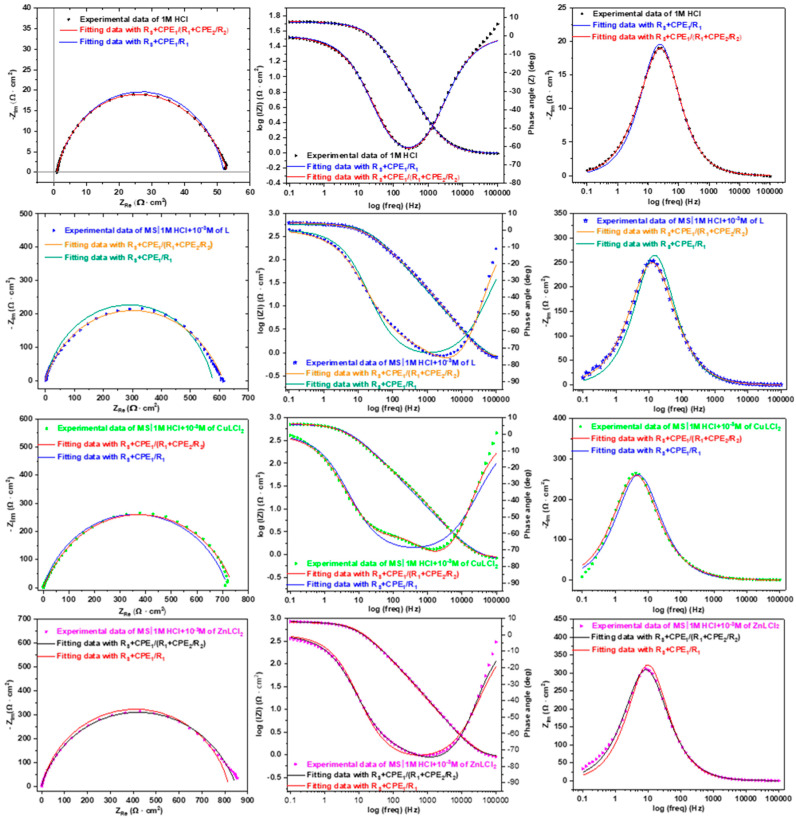
Fitting comparison between R_s_ + CPE_1_/R_1_ and R_s_+CPE_1_/(R_1_+CPE_2_/R_2_).

In addition, Figure 4 presents both the experimental results and their corresponding simulations. Additionally, a third representation, showing the evolution of the imaginary part as a function of frequency, was adopted to clearly illustrate the quality of the fitting, as the imaginary part is particularly sensitive to variations in frequency. It is clearly apparent that a better fit was achieved with the circuit model with two time constants. Even in the case of L and HCl, which show only one minimum in the phase angle plot, the interface response indicates a more complex behavior. Specifically, it suggests the involvement of a second elementary step in the overall process, which begins to have a role before the relaxation of the first step is completed. This implies that the reaction of inhibitors with the interface involves two phenomena that occur simultaneously, particularly near the frequency of 100 Hz. As a result, modeling the system with more than one time constant becomes essential to accurately capture and understand the full scope of the interfacial processes [16].

Table 1 summarizes the fitting results obtained using the circuit model with two time constants. In this model, *R*_s_ represents the solution resistance, which is in series with the response of the first-capacitive-resistive phenomenon. This phenomenon is modeled using a constant phase element (CPE) in parallel with a resistance that can be attributed to the rapid relaxation of the double layer at the electrochemical interface, which occurs in parallel with charge transfer. The components *CPE*_dl_ and *R*_ct_ correspond, respectively, to the capacitive and charge transfer resistance behavior of this fast process, which occurs at high frequencies. The second time constant is often attributed to the response of the interface following the adsorption of the inhibitor to the surface of the MS. This layer also exhibits capacitive-resistive behavior and is modeled using a constant phase element *CPE*_ads_ in parallel with adsorption resistance R_ads_. The adsorption process involves the inhibitors creating a protective barrier that affects the interface, particularly at lower frequencies, where the effects of this layer are more pronounced.

The EIS fitting parameters clearly demonstrate the enhanced corrosion protection offered by the ligand L and its metal complexes CuLCl_2_ and ZnLCl_2_ when compared to the uninhibited 1.0 M HCl solution. The *R*_s_ remains nearly constant across all systems, confirming that observed changes originate from interfacial phenomena. Upon inhibitor addition, the CPE parameter of double-layer capacitance (*Q*_1_ = *Q*_dl_) decreases significantly from 271.6 μF s^n−1^ cm^−2^ in HCl to as low as 6.55 μF s^n−1^ cm^−2^ for L, indicating effective surface coverage by the inhibitor molecules and a reduction in active surface area. This is supported by higher n_1_ values (approaching 1), suggesting more homogeneous interfacial behavior [35]. The *R*_1_ = *R*_ct_ notably increases with inhibitor presence, with ZnLCl_2_ reaching 169.4 Ω·cm^2^, reflecting a strong suppression of electrochemical reactions. Simultaneously, the *Q*_2_ = *Q*_ads_ drops dramatically in all inhibited systems, while the film resistance (*R*_2_ = *R*_ads_) rises sharply, reaching over 680 Ω·cm^2^ for both CuLCl_2_ and ZnLCl_2_, confirming the formation of stable and resistive protective films. Consequently, the total polarization resistance (*R*_p_) increases from 52.03 Ω·cm^2^ in 1.0 M HCl to 845.70 Ω·cm^2^ with ZnLCl_2_, resulting in high inhibition efficiencies (*η*_EIS%_) of 91.5%, 92.9%, and 93.8% for L, CuLCl_2_, and ZnLCl_2_, respectively. These results confirm that complexation with Zn and Cu significantly enhances the performance of the benzimidazole–thiophene ligand, with ZnLCl_2_ exhibiting the most effective barrier properties. The low χ^2^/|Z| values across all fits further validate the accuracy of the equivalent circuit model and the reliability of the extracted parameters

**Figure 5:**
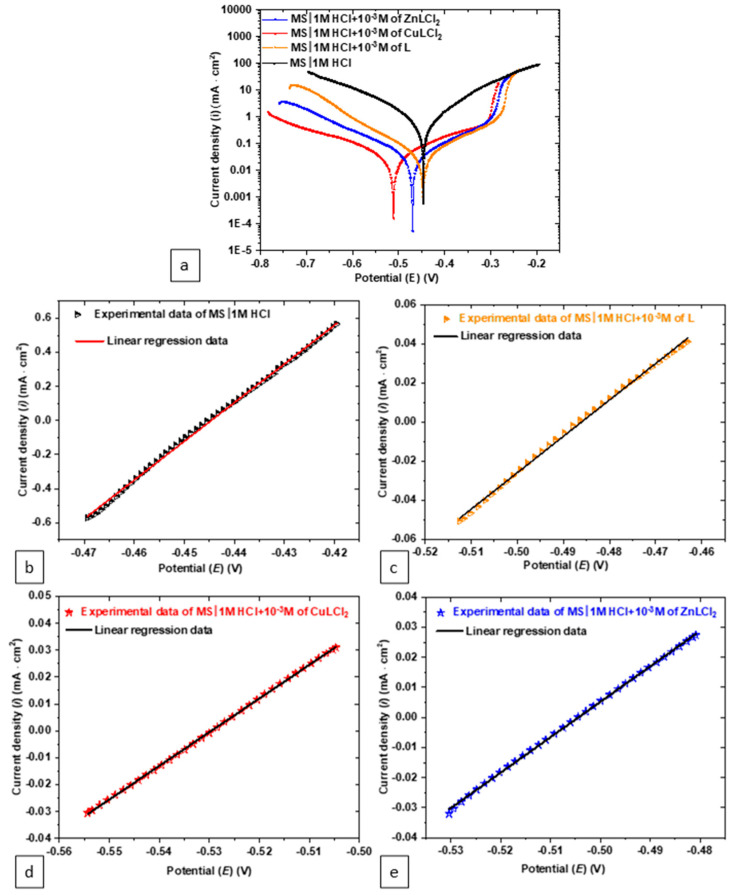
Polarization test results: (**a**) PDP graph and (**b**–**e**) LPR graphs.

The PDP and LPR techniques were employed to study the effect of adding compound L and its complexes into HCl solution on the MS surface. As illustrated in Figure 5, the addition of the three compounds to the aggressive HCl solution shifted the polarization curves sharply downward, positioning them below the curve for HCl alone. This shift indicates a significant reduction in corrosion activity [36]. The observed behavior confirms that the compounds were adsorbed onto the MS surface, forming a protective barrier layer. This barrier effectively inhibits the interaction between the surface and the corrosive ions in the acidic solution, reducing the rate of metal dissolution. In addition, a significant decrease in cathodic currents is also observed, indicating that H+ reduction is limited by inhibitor adsorption and therefore inhibits MS electrode corrosion [37]. On the anodic side, when the potential exceeded −0.3 V, a rapid increase in current was observed, indicating a significant change in the surface interactions. At this potential, all the polarization curves, regardless of the compound added, merged with the curve of HCl alone. This phenomenon suggests that the protective compounds were desorbed because of the high anodic potential [38]. The potential at which this desorption occurs is referred to as the desorption potential, marking a critical point where the effectiveness of the inhibitors drops under anodic polarization [39].

The electrochemical parameters regrouped in Table 2, including *E*_corr_, *i*_corr_, Tafel slopes, and *R*_p_, were extracted from the PDP curves and LPR measurements using Tafel fitting and linear regression, respectively. The comparison between Tafel curves and linear polarization resistance LPR reveals consistent trends in evaluating the corrosion inhibition performance of the tested compounds, though some differences in the *i*_corr_ values are observed. Tafel polarization provides a detailed analysis of both anodic and cathodic reaction kinetics, offering direct insights into corrosion mechanisms [40]. For instance, in the HCl solution alone, *i*_corr_ from Tafel fitting is 1059.37 µA/cm^2^, while for the ZnLCl_2_ complex, it decreases significantly to 33.22 µA/cm^2^, confirming its superior inhibition efficiency. LPR, in contrast, simplifies the evaluation by deriving polarization resistance (*R*_p_), which inversely correlates with the corrosion rate [40,41,42]. The Rp values further support the Tafel results, with the ZnLCl_2_ complex showing the highest *R*_p_ (850 Ω·cm^2^) compared to the CuLCl_2_ complex (615 Ω·cm^2^) and the ligand alone (552 Ω·cm^2^), reinforcing the conclusion that the ZnLCl_2_ complex provides the most effective protection.

The CASP method was employed to offer a complementary perspective by analyzing the response of an electrochemical system subjected to sinusoidal modulation of constant amplitude and frequency [43]. When the solution resistance is negligible in comparison to the polarization resistance, the relationship between current and potential at low frequencies can be expressed by the following Equation (2):

(2) $$I\left(t\right)=I_{corr}\left(exp(\frac{E\left(t\right)-E_{corr}}{\beta_{a}/ln10})-exp(-\frac{E\left(t\right)-E_{corr}}{\beta_{c}/ln10})\right),$$

When the electrode potential is modulated sinusoidally around the corrosion potential, with an amplitude Va and a frequency fs, its expression follows Equation (3):

(3) $$E\left(t\right)=E_{corr}+V_{a}sin(2\pi f_{s}t)E(t),$$

Equation (2) becomes Equation (4) as follows:

(4) $$I\left(t\right)=I_{corr}\left(exp(\frac{V_{a}\;sin(2\pi f_{s}t)}{\beta_{a}/ln10})-exp(-\frac{V_{a}\;sin(2\pi f_{s}t)}{\beta_{c}/ln10})\right),$$

**Figure 6:**
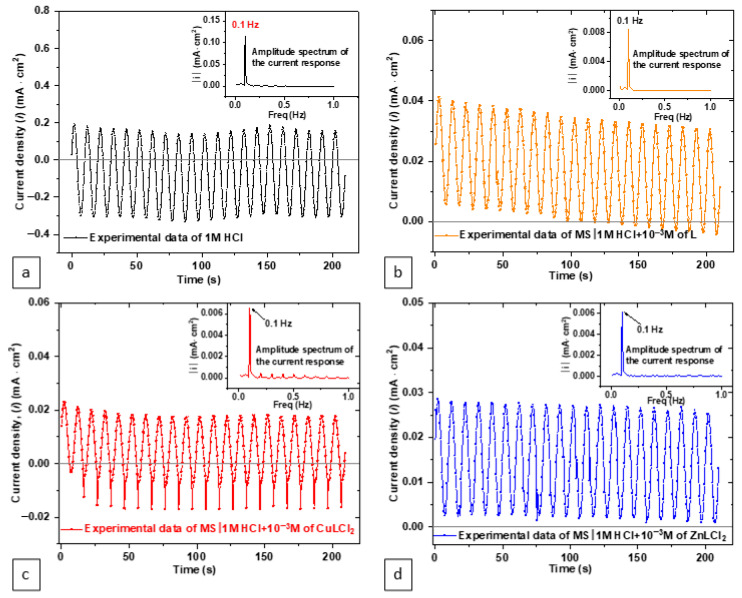
Time-dependent current response obtained from CASP measurements for MS (**a**) in 1.0 M HCl, (**b**) in 1.0 M + 10^−3^ M L, (**c**) in 1.0 M + 10^−3^ M CuLCl_2_, and (**d**) in 1.0 M + 10^−3^ M ZnLCl_2_.

The resulting current response is a periodic signal with a period identical to that of the sinusoidal modulation. Expressed as the sum of sinusoidal components at the fundamental frequency of the electrode potential modulation, as well as its harmonics integer multiples of that frequency [44]. The result is the current vs. time curve shown in Figure 6, corresponding to the modulation of the electrode voltage in the same figure, for a frequency of 0.1 Hz (Figure insert). The CASP calculation tool integrated into the EC-Lab software performs a Discrete Fourier Transform (DFT) of the current vs. time response, extracts the amplitudes of the harmonic components, and subsequently calculates the key corrosion parameters: *i*_corr_, *β*_a_, and *β*_c_ [26,45]. CASP-derived *i*_corr_ values also follow the same decreasing trend, with HCl at 404.5 µA/cm^2^, ZnLCl_2_ at 21.83 µA/cm^2^, CuLCl_2_ at 37.68 µA/cm^2^, and L at 30.33 µA/cm^2^. The CASP, Tafel, and LPR methods reveal the same trend, although the CASP values for *i*_corr_ are generally lower than those from Tafel fitting justifying the destructive nature of the PDP technique [26]. This difference arises because CASP captures the overall system behavior, considering both kinetic and capacitive effects, whereas Tafel polarization emphasizes reaction kinetics more specifically.

The comparative study of these three methods highlights their respective strengths and limitations. Tafel polarization excels in providing mechanistic insights into anodic and cathodic reactions, but its sensitivity to experimental conditions and assumptions in Tafel fitting may lead to slight discrepancies in *i*_corr_ values. LPR offers a straightforward and robust measure of *R*_p_, but it lacks the detailed kinetic resolution provided by Tafel. CASP, as a dynamic technique, bridges these approaches by combining kinetic and capacitive responses, making it an effective tool for confirming trends and providing additional context [46]. The agreement among the three methods underscores the reliability of the findings, with each method complementing the others to provide a comprehensive evaluation of the inhibitors’ performance. Overall, this multi-technique approach highlights the superior inhibition efficiency of the ZnLCl_2_ complex, followed by the CuLCl_2_ complex and the ligand alone, in protecting mild steel against corrosion in HCl.

### 3.2. Analysis of Corrosion Products and Protective Layer

**Figure 7:**
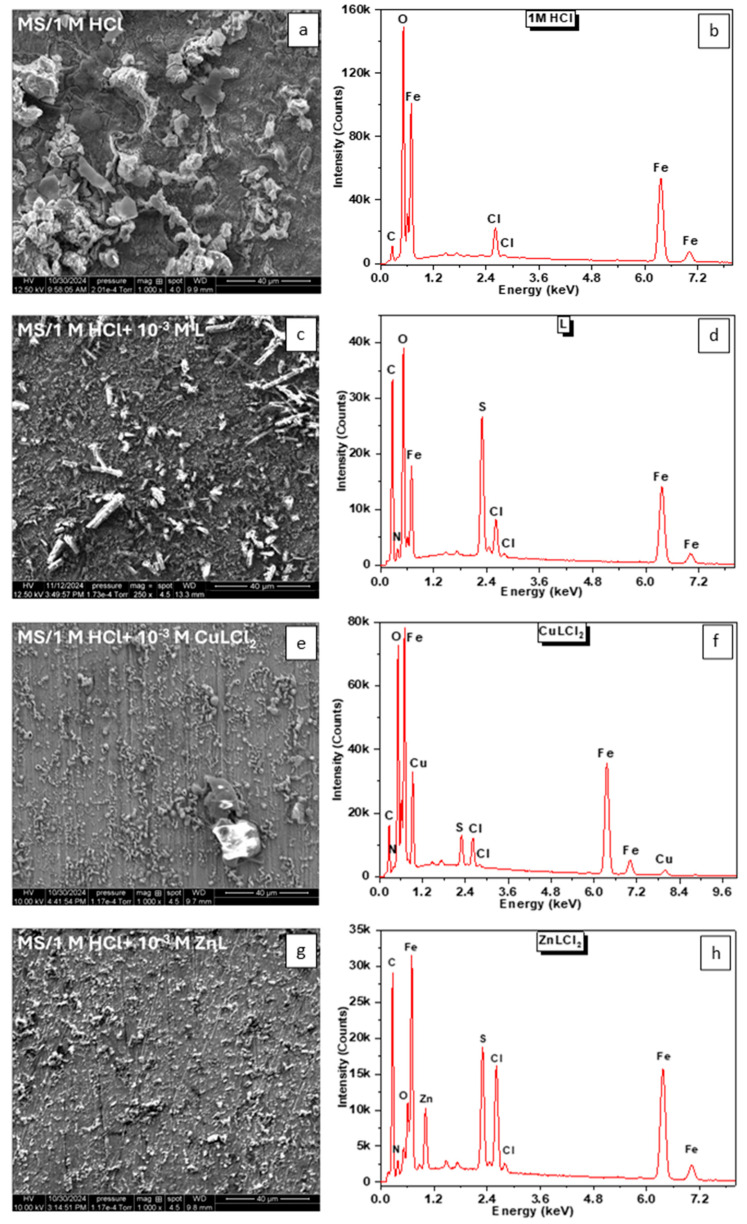
SEM images and EDS spectra MS samples after immersion in 1.0 M HCl without and with inhibitors: (**a**,**b**) MS in 1.0 M HCl; (**c**,**d**) with 10^−3^ M L; (**e**,**f**) with 10^−3^ ZnLCl_2_; (**g**,**h**) with 10^−3^ CuLCl_2_.

Figure 7 shows corrosion morphologies in the absence and presence of various inhibitors based on SEM observations. Corrosion products are more abundant after the metal has been corroded in 1.0 M HCl solution indicating that the metal has been severely corroded (Figure 7a). In the presence of the three inhibitors, MS surface was gradually covered by adsorbed layer (Figure 7c,e,g). In addition, the differences in surface states result from the unique interaction mechanisms of each inhibitor with the MS surface. These interactions depend on the chemical structure, adsorption capacity, and specific functional groups of the inhibitors. For instance, compounds of ZnLCl_2_ and CuLCl_2_ are likely to form more stable adsorbed layers due to the synergistic effects of the metal ions and the organic ligand. In contrast, the ligand alone may rely solely on its organic structure for adsorption, leading to a comparatively weaker or less uniform protective film. This distinct interaction directly affects corrosion inhibition efficiency which explains variations in electrochemical parameters such as *R*_p_ (EIS).

The EDS results reveal that all elements present in the structure of the ligand and its complexes were adsorbed onto the MS surface. This observation confirms that the inhibition efficiency is not solely dependent on a single component but rather arises from a synergistic effect of all the atoms in the chemical composition of the compounds.

**Figure 8:**
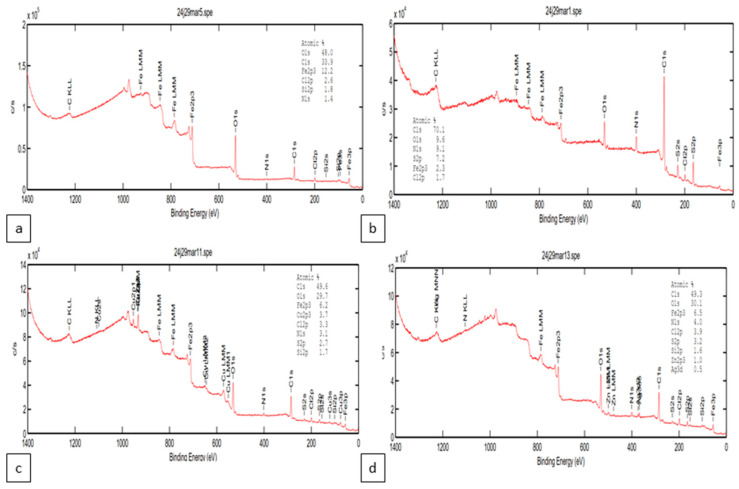
XPS survey spectra of the sample surfaces after immersion in 1.0 M HCl without and with inhibitors: (**a**) MS in 1.0 M HCl; (**b**) with 10^−3^ M L; (**c**) with 10^−3^ ZnLCl_2_; (**d**) with 10^−3^ CuLCl_2_.

The chemical composition of non-inhibited and inhibited interfaces was determined by XPS analyses after immersion of MS in 1.0 M HCl with and without the optimum concentration of inhibitors. Figure 8 represents the four survey spectra which confirms the EDS results, and all the elements present in the structure of inhibitors are founded in the MS surface as well as the aggressive ions of the solutions like Cl^−^.

**Figure 9:**
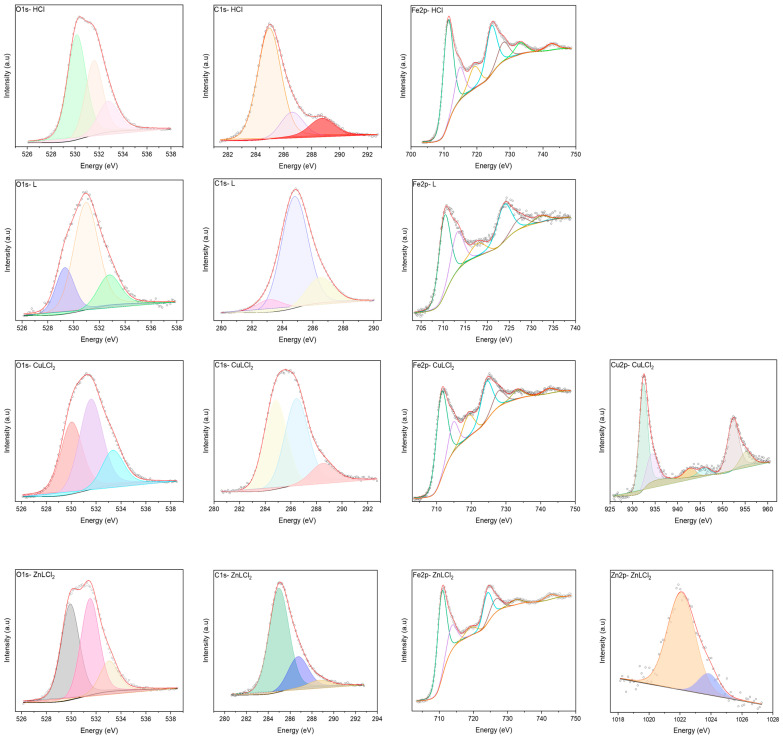
High-resolution spectra of O 1s, C 1s, Fe 2p, Cu 2p and Zn 2p of the four surfaces mentioned in Figure 8.

The high-resolution spectra of C 1s, O 1s, Fe 2p, Cu 2p and Zn 2p were analyzed with deconvolution fitting (Figure 9). The binding energies (BE, eV) as well as the corresponding assignment of each peak are given in Table 3. The XPS spectrum of the Fe 2p reveals key insights into the chemical states and surface composition of MS exposed to HCl solution with and without inhibitors. All XPS spectra show the same bending energy in the presence of inhibitors compared to the surface exposed to HCl alone, with a slight shift in some cases. The Fe 2p_3/2_ main peak at 711.21 eV is characteristic of Fe^3+^ species, commonly associated with iron oxides and hydroxides such as Fe_2_O_3_ or FeOOH [47,48,49,50,51]. The presence of a peak at 714.77 eV represents a shake-up satellite, which is typically observed in Fe^3+^ compounds due to multiplet splitting, confirming the high-spin state of Fe^3+^ [50,51,52]. Another peak at 719.18 eV is also attributed to a higher-order satellite feature, reinforcing the dominant Fe^3+^ presence [50,51,52]. For Fe 2p_1/2_ region, the primary peak at 724.38 eV corresponds to Fe^3+^ [51,52,53], while the peak at 727.98 eV is another shake-up satellite, further supporting the presence of oxidized Fe species [50,51,53]. The peak at 733.10 eV represents an additional satellite feature, often observed in Fe^3+^ systems, particularly in hydrated or complex iron species [50,51,52]. The high binding energy peak at 742.52 eV likely corresponds to an extended satellite feature, confirming the presence of a strong Fe^3+^ chemical environment [50,51,52].

The XPS analysis of the O 1s provides a peak at ~530 eV that corresponds to lattice oxygen (O^2−^) in metal oxides, confirming the formation of a stable oxide layer within all cases [54]. The peak at ~531 eV is attributed to hydroxyl groups (-OH), indicating the presence of metal hydroxides [49]. Additionally, the peak appears around 532 eV corresponds to adsorbed water (H_2_O) or oxygenated species, suggesting the presence of hydrated corrosion products or chemisorbed oxygen [55].

The XPS profile of the C 1s provides valuable insights into the adsorption behavior of organic inhibitors on the iron surface in an HCl environment. In the absence of an inhibitor, the spectrum primarily exhibits peaks at ~284 eV, 286 eV, and 288 eV, corresponding to adventitious carbon (C–C, C–H), oxygen-containing carbon species (C–O, C–OH), and carboxyl or carbonate groups (O–C=O), respectively [56,57,58]. These peaks suggest that the surface contains residual organic contamination and oxidation products. Upon introducing the organic inhibitor (L), the peak at 286.60 eV becomes more pronounced, indicating the adsorption of functional groups such as C–O and C–N, which are likely part of the inhibitor’s molecular structure. This confirms that the inhibitor interacts with the metal surface, forming a protective layer. The presence of a Cu-based inhibitor (CuL) results in noticeable changes, particularly in the 286–288 eV range, where new peak components emerge, suggesting Cu–ligand interactions (Cu–N, Cu–O), indicative of chemisorption [56]. Similarly, the Zn-based inhibitor (ZnL) shows an increase in intensity at 288.75 eV, suggesting the formation of Zn–O or Zn–N coordination bonds, as well as possible carbonate species, which could play a role in corrosion inhibition [59]. These findings highlight the role of metal–ligand coordination in enhancing inhibitor efficiency, likely contributing to improved corrosion protection in an acidic environment.

The XPS analysis of the Cu 2p reveals the presence of multiple oxidation states of copper in an HCl environment with an organic inhibitor. The peak at 932.40 eV corresponds to Cu^+^ (Cu_2_O, cuprous oxide), indicating that a portion of copper remains in a reduced state [60,61]. In contrast, the peak at 934.27 eV is characteristic of Cu^2+^ species, typically associated with CuO (cupric oxide) or Cu(II) complexes, suggesting partial oxidation [60,61]. The presence of shake-up satellite peaks at 942.69 eV and 946.00 eV further confirms the existence of Cu^2+^, as these features arise from charge transfer effects in high-spin Cu^2+^ compounds [62]. Similarly, in the Cu 2p_1/2_ region, the main peak at 952.29 eV corresponds to a mix of Cu^+^, and Cu^2+^, while the satellite peak at 955.16 eV reinforces the presence of Cu^2+^ oxidation states [62]. The coexistence of Cu^+^ and Cu^2+^ suggests that copper undergoes partial oxidation, likely forming Cu_2_O and CuO on the surface, with Cu^2+^ interacting with chloride ions in solution.

The XPS analysis of the Zn 2p shows binding energy values at 1022.08 eV and 1023.85 eV [63]. The first peak is characteristic of Zn^2+^ species, commonly associated with zinc oxides (ZnO), zinc hydroxides (Zn(OH)_2_), or zinc complexes formed through interactions with the inhibitor [63,64]. This confirms that zinc exists predominantly in its oxidized state rather than in a metallic (Zn) form. The peak at 1023.85 eV suggests the presence of zinc-organic complexes or ZnCl_2_, indicating that Zn^2+^ interacts with chloride ions from HCl or functional groups in the inhibitor, such as oxygen or nitrogen-containing species [64,65]. In addition, the appearance of peaks of N and S in protected sample surface spectra (Figure 8) confirms the adsorption L also the metal on the iron surface.

### 3.3. Corrosion Inhibition Mechanisms

Among the tested compounds, the zinc complex exhibited superior inhibition efficiency compared to the copper complex, a trend consistently observed across all electrochemical and surface characterization techniques. This enhanced performance can be attributed to several factors. First, Zn^2+^ forms more stable and compact complexes with the benzimidazole–thiophene ligand through coordination with nitrogen and sulfur donor atoms, resulting in a highly planar structure that favors dense and uniform adsorption onto the mild steel surface [66,67]. In contrast, Cu^2+^ complexes are often characterized by distorted geometries and greater lability, which may lead to less homogeneous surface coverage [68,69]. Furthermore, Zn ions are known to promote the formation of insoluble Zn-based species such as Zn(OH)_2_ or ZnO in acidic environments, which may reinforce the inhibitor layer and enhance passivation. Copper ions, on the other hand, can undergo redox cycling (e.g., Cu^2+^/Cu^+^), potentially destabilizing the protective film and even accelerating localized corrosion under certain conditions [70]. These mechanistic insights are supported by XPS and EDS results, which confirmed stronger surface retention and interaction of the Zn complex compared to Cu, validating its superior performance as a corrosion inhibitor in this system.

## 4. Conclusions

This work presents a comprehensive investigation into the corrosion inhibition performance of a novel thiophene–benzimidazole-based ligand and its zinc and copper complexes for mild steel in acidic media. The integration of electrochemical techniques including EIS, Tafel polarization, LPR, and the advanced CASP method, demonstrated that all compounds significantly reduced the corrosion rate, with the Zn complex exhibiting the highest inhibition efficiency, reaching up to 93.8%. CASP analysis provided dynamic insights into the corrosion process, confirming trends observed in steady-state measurements.

Surface characterization via SEM and EDS revealed smoother morphologies and inhibitor-derived elemental signals on protected samples, while XPS confirmed the formation of Fe^3+^-rich oxides and strong interaction between the inhibitor molecules and the metal surface.

The superior performance of the Zn complex compared to its Cu analog is attributed to the greater stability and planarity of the Zn–ligand complex, which favors more uniform surface adsorption. Additionally, Zn^2+^ ions promote the formation of insoluble passive species that reinforce the protective barrier, while Cu^2+^ may participate in redox cycling, potentially compromising the integrity of the inhibition film. This mechanistic distinction was confirmed by surface analyses, which showed stronger retention and more stable interaction of the Zn complex with the steel surface.

Overall, this study highlights the potential of metal-complexed benzimidazole-based systems as efficient, environmentally friendly corrosion inhibitors and underscores the importance of combining multi-technique electrochemical and surface analyses to unravel their inhibition mechanisms. The insights gained here provide a foundation for the rational design of advanced inhibitors tailored to industrial applications in aggressive environments.

## Figures and Tables

**Table 1:** EIS parameters derived from the interface MS/1.0 M HCl in the absence and presence of inhibitors L, CuLCl_2_ and ZnLCl_2_ and obtained with 2RQ model.

MS/1.0 M HCl + Inhibitor System	1.0 M HCl	L	CuLCl_2_	ZnLCl_2_
*R*_S_ (Ω cm^2^)	0.98	0.79	0.86	0.88
*Q*_1_ (μF s^n−1^ cm^−2^)	271.6	6.55	22.74	20.76
n_1_	0.844	0.98	0.93	0.90
*R*_1_ (Ω cm^2^)	47.62	50.59	55.61	169.4
*Q*_2_ (μF s^n−1^ cm^−2^)	17670	59.3	95.46	49.91
n_2_	0.71	0.67	0.72	0.67
*R*_2_ (Ω cm^2^)	4.41	564	686.9	681.3
*R*_p_ (Ω cm^2^)	52.03	614.59	742.51	845.70
***η***_EIS%_	-	91.5	92.9	93.8
χ^2^/|Z|	0.058	0.094	0.119	0.106

**Table 2:** Comparative parameters derived from CASP, Tafel and LPR measurements.

Specimen	CASP				PDP—Tafel Fit					LPR—R_p_ Fit			
	*i_corr_*	*β_a_*	*|β_c_|*	*R_p-calc_*	*E_corr_*	*i_corr_*	*β_a_*	*|β_c_|*	*R_p- calc_*	*E_corr_*	*R_p_*	*i_corr- calc_*	*R^2^*
HCl 1.0 M	404.5	78.5	77.9	42	−447.3	1059.4	120.5	147.5	27.18	−444.6	44	386.2	0.99
L	30.3	70.9	76.3	526.2	−453.6	33.8	99.6	107.9	665.75	−482.8	552	28.9	0.99
CuLCl_2_	37.7	108.7	109.4	628.3	−506.6	60	218.3	191.8	738.99	−512.2	615	38.5	0.99
ZnLCl_2_	21.8	80.2	78.8	790.9	−468.5	33.22	129.2	134.2	860.41	−504.4	850	18.8	0.99

Note: *E*_corr_ in mVAg/AgCl, 3M KCl, *i*_corr_ in µA cm^−2^, *β*a and *β*c in mV/dec and *R*_p_ in Ω cm^2^.

**Table 3:** Binding energy and corresponding assignment.

Specimen	Element	Binding Energy (eV)
1.0 M HCl	O	530.13–531.58–532.77
	C	284.97–286.60–288.75
	Fe	711.21–714.77–719.18–724.38–727.98–733.10–742.52
L	O	529.32–530.96–532.77
	C	283.16–284.81–286.48
	Fe	710.29–713.17–717.82–723.79–727.49–732.13
CuLCl_2_	O	530.03–531.58–533.34
	C	284.81–286.43–288.52
	Fe	711.42–714.78–719.18–724.38–727.89–733.31–742.45
	Cu	932.40–934.27–942.69–946.0–952.29–955.16
ZnLCl_2_	O	529.91–531.51–533.04
	C	284.98–286.74–288.71
	Fe	710.74–713.61–719.18–724.05–726.46–732.32–742.45
	Zn	1022.08–1023.85

## Data Availability

The original contributions presented in this study are included in the article. Further inquiries can be directed at the corresponding author.
